# Breeding Strategy Determines Rupture Incidence in Post-Infarct Healing WARPing Cardiovascular Research

**DOI:** 10.1371/journal.pone.0139199

**Published:** 2015-09-25

**Authors:** Sophie Deckx, Paolo Carai, John Bateman, Stephane Heymans, Anna-Pia Papageorgiou

**Affiliations:** 1 Department of Cardiology, Maastricht University, Maastricht, The Netherlands; 2 Centre for Molecular and Vascular Biology, KULeuven, Leuven, Belgium; 3 Murdoch Children’s Research Institute, University of Melbourne, Royal Children’s Hospital, Parkville, Victoria, Australia; 4 Department of Paediatrics, University of Melbourne, Royal Children’s Hospital, Parkville, Victoria, Australia; University of Buenos Aires, Faculty of Medicine. Cardiovascular Pathophysiology Institute., ARGENTINA

## Abstract

**Background:**

Von Willebrand A domain Related Protein (WARP), is a recently identified extracellular matrix protein. Based upon its involvement in matrix biology and its expression in the heart, we hypothesized that WARP regulates cardiac remodeling processes in the post-infarct healing process.

**Methods and results:**

In the mouse model of myocardial infarction (MI), WARP expression increased in the infarcted area 3-days post-MI. In the healthy myocardium WARP localized with perlecan in the basement membrane, which was disrupted upon injury. *In vitro* studies showed high expression of WARP by cardiac fibroblasts, which further increases upon TGFβ stimulation. Furthermore, WARP expression correlated with aSMA and COL1 expression, markers of fibroblast to myofibroblast transition, *in vivo and in vitro*. Finally, WARP knockdown *in vitro* affected extra- and intracellular basic fibroblast growth factor production in myofibroblasts. To investigate the function for WARP in infarction healing, we performed an MI study in WARP knockout (KO) mice backcrossed more than 10 times on an Australian C57Bl/6-J background and bred in-house, and compared to wild type (WT) mice of the same C57Bl/6-J strain but of commercial European origin. WARP KO mice showed no mortality after MI, whereas 40% of the WT mice died due to cardiac rupture. However, when WARP KO mice were backcrossed on the European C57Bl/6-J background and bred heterozygous in-house, the previously seen protective effect in the WARP KO mice after MI was lost. Importantly, comparison of the cardiac response post-MI in WT mice bred heterozygous in-house versus commercially purchased WT mice revealed differences in cardiac rupture.

**Conclusion:**

These data demonstrate a redundant role for WARP in the wound healing process after MI but demonstrate that the continental/breeding/housing origin of mice of the same C57Bl6-J strain is critical in determining the susceptibility to cardiac rupture and stress the importance of using the correct littermate controls.

## Introduction

Ischemic heart disease is one of the most prominent cardiovascular diseases (CVD) and can lead to sudden death or to heart failure (HF). Despite a decline in death rates, the burden of CVD remains high, with over 5 million people suffering from HF in the United States [1]. Therefore, it is critical to identify novel pathogenic mechanisms for designing new treatment strategies, in order to prevent the progression of this disease. The cardiac response after MI is a tightly regulated and well-orchestrated process of wound healing. Following an ischemic event, there is rapid formation of granulation tissue, a tissue rich in leukocytes, vessels and proliferating fibroblasts. This granulation tissue regresses and is replaced by collagenous matrix proteins, which will constitute the mature scar tissue [2]. Disturbances in this healing response lead to adverse infarct healing, cardiac rupture and HF. Our group has previously demonstrated that the matrix reinforcing capacities of the non-structural proteins Thrombospondin-2, Osteonectin and Osteoglycin are critical in coping with increased loading [3], advanced aging [4] or ischemia [5, 6] of the heart.

WARP, a small, non-collagenous, secreted glycoprotein, was recently identified and shown to be expressed in the extracellular matrix of the heart. WARP contains a von Willebrand factor type A domain [7] and the expression of WARP is restricted to permanent cartilages and to basement membranes of peripheral nerves, skeletal and cardiac muscle and the central nervous system vasculature [8]. Interestingly, in basement membranes it interacts with perlecan, a protein important for stability and critical during cardiac development as well as during wound healing after MI [9–12]. WARP interacts with the heparan sulphate chains containing domain I of perlecan, where also the interaction of basic fibroblast growth factor (bFGF) and vascular endothelial growth factor (VEGF) take place [13], growth factors essential during infarct healing [14–20]. Furthermore, WARP interacts with collagen VI [21, 22], a known HF- related gene [3, 21, 22]. Collectively, these studies support the hypothesis that WARP is needed during infarct healing and cardiac remodeling.

Using *in vitro* and *in vivo* models, we assessed the role for WARP during the different remodeling phases after MI. Our results were unexpectedly overshadowed by the influence of the breeding conditions of the WARP KO and WT mice of the same C57Bl6-J strain. The use of genetically manipulated mice is a widespread tool to study the effects of a specific gene in cardiac remodeling. The importance of the genetic background of the inbred strain used in the generation and analysis of transgenic and KO animals [23–26] has been recognized but little is known about the regional differences within the same strain that affect susceptibility to CVD in mice. Our data demonstrate that the breeding conditions (such as genetic background, continental source and housing) are critical factors that determine the susceptibility to CVD and stress the importance of using the correct littermate controls. Finally, though WARP is highly expressed by cardiac fibroblasts and is associated with the activation of myofibroblasts *in vivo* and *in vitro*, it does not appear to play a critical role in the wound healing following MI.

## Results

### WARP expression is induced during cardiac remodeling

**Fig 1:**
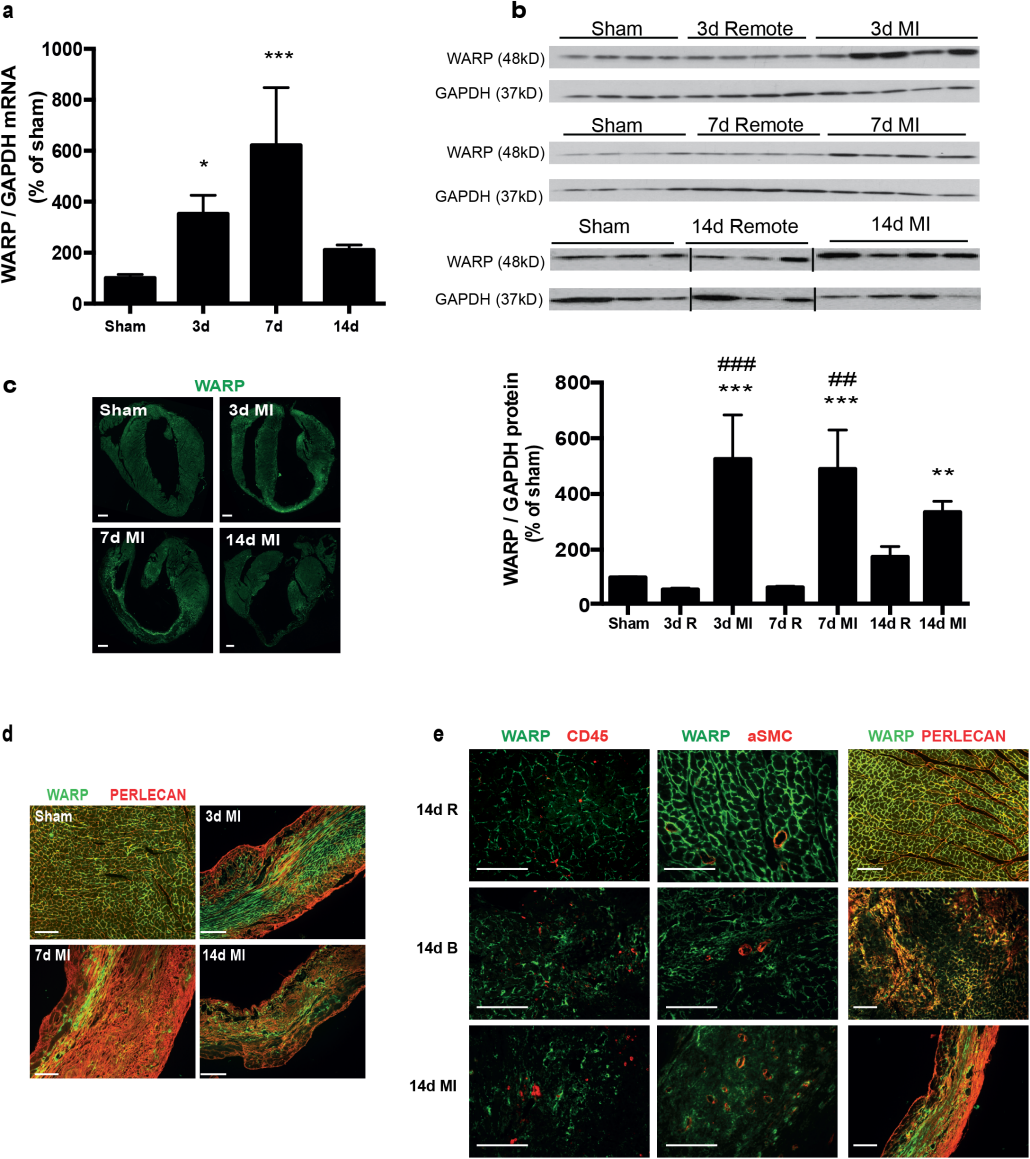
WARP expression pattern in the heart. (a) WARP mRNA levels were induced 3 and 7 days after MI. WARP mRNA levels peaked at 7 days after MI and then decreased again at 14 days. (b) Western blot analysis and (c) immunohistochemistry also showed an induction of WARP protein levels in the infarcted LV at 3, 7, and 14 days after MI. Because of different sample-loading, the blots of the 14 days MI were cut and pasted in the same order as the blots of the 3 and 7 days MI. Images of the original unadjusted blots are provided in S1 Fig (d and e) Confocal microscopy confirmed the co-localization of WARP with perlecan in the uninfarcted heart, showing a network of WARP and perlecan as a honeycomb-structure surrounding the cardiomyocytes. In the infarcted LV zones, the WARP-perlecan honeycomb-structure is reduced at 3 days after MI and completely absent at 7 and 14 days after MI and the interaction between WARP and perlecan is disrupted. (e) WARP did not localize on CD45 positive leukocytes or in alpha smooth muscle cells lining the vessels in the remote, border and infarcted zone of the heart 14 days after MI. n≥3; *p≤0.05; **p≤0.005; ***p≤0.001 versus sham, ##p≤0.005; ###p≤0.001 versus remote, bars 1000 μm for (c) and 100 μm for (d) and (e).

To assess the role of WARP during the different phases of cardiac remodeling after MI, we executed a time-series of a mouse model of permanent coronary occlusion in C57Bl/6-J WT mice, and examined WARP levels in left ventricle (LV) tissue samples of sham-operated mice and of mice 3, 7 and 14 days after MI (Fig 1). Real-time PCR showed an induction of WARP gene expression after MI starting already at 3 days post ligation. WARP mRNA levels peaked 7 days after MI, and decreased back at 14 days, but not completely back to baseline levels (Fig 1A). Western blot analysis of WARP protein expression and immunohistochemistry showed a similar induction of WARP protein levels after MI in the infarcted LV, significantly increasing at 3 and 7 days post ligation and then slightly decreasing again at 14 days, but not back to baseline levels (Fig 1B and 1C). Confocal microscopy revealed a unique pattern for WARP in the infarcted heart. In the un-infarcted regions of the LV, WARP is localized in the extracellular matrix and in line with previous reports, WARP co-localized with perlecan [9], a marker for the basement membrane, showing a network of WARP and perlecan as a honeycomb-structure surrounding the cardiomyocytes (Fig 1D). However, in the infarcted LV, this honeycomb- structure is reduced at 3 days and completely absent at 7 and 14 days after MI and importantly, the interaction between WARP and perlecan is disrupted (Fig 1D). The early increased expression of WARP mRNA and protein levels is in parallel with the influx of inflammatory cells in the damaged myocardium and with the formation of the granulation tissue post infarction [2]. However, co-staining of WARP and CD45-postitive leukocytes revealed no localization of WARP on inflammatory cells (Fig 1E). Furthermore, WARP did not co-localize with the alpha smooth muscle actin of the vessels (Fig 1E). These results suggest a function for WARP in scar formation but not during inflammation.

### WARP expression correlates with fibroblast to myofibroblast transition and regulates bFGF levels

**Fig 2:**
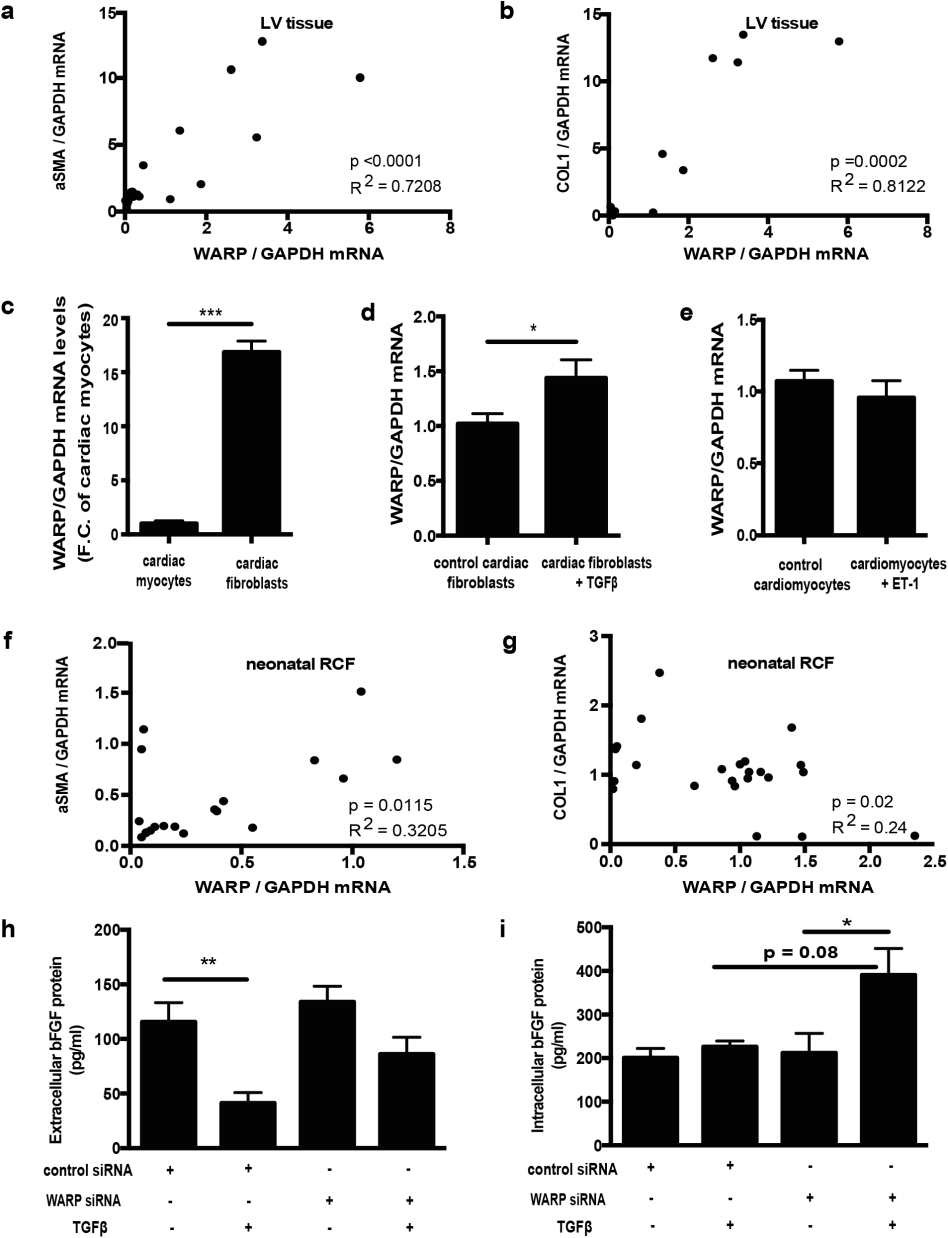
WARP expression correlates with myofibroblast transformation and affects cardiac bFGF levels (a) WARP mRNA levels correlate with aSMA and (b) COL1 levels in infarcted LV tissue. (c) WARP mRNA levels are 17 fold higher in neonatal rat cardiac fibroblasts compared to cardiac myocytes. (d) Treatment of the cardiac fibroblasts with TGFβ, a known pro-fibrotic stimulus, increased WARP expression, (e) while there was no change in WARP expression after treatment of the cardiomycoytes with Endothelin-1. (f) In isolated cardiac fibroblasts WARP expression correlates with aSMA and (g) COL1 expression. (h) TGFβ stimulation of scrambled control siRNA treated fibroblasts caused a significant decrease in extracellular bFGF protein levels, (i) whilst intracellular bFGF levels did not change. WARP knockdown alone did not affect extra-or intra-cellular bFGF levels but when cells were stimulated with TGFβ under knockdown conditions there was no significant decrease in extracellular bFGF levels anymore and a trend to increased intracellular bFGF levels was seen in the WARP-siRNA treated cells (p = 0.08). n≥3; *p<0.05; ***p<0.001; ****p<0.0001.

Because the early increased expression of WARP post-infarction is in parallel with the transition of cardiac fibroblasts into myofibroblasts and with the formation of the granulation tissue post infarction, we correlated WARP expression levels in the infarcted LV tissue with α- smooth muscle actin (aSMA) and collagen I (COL1) expression levels, 2 elements that mark the conversion of fibroblast to myofibroblast [27]. We found highly significant correlations of WARP with these markers (Fig 2A and 2B), indicating a possible role for WARP in myofibroblast transformation.

When *in vitro* WARP expression levels were measured in un-stimulated neonatal rat cardiac fibroblasts and cardiomyocytes, we found a 17 fold higher WARP expression in cardiac fibroblasts as compared to cardiomyocytes (Fig 2C). Furthermore, WARP expression in cardiac fibroblasts increased significantly after TGFβ stimulation (Fig 2D), a known pro-fibrotic growth factor that stimulates the conversion of fibroblast to myofibroblast following MI [20] whereas Endothelin-1 stimulation (an inducer of cardiomyocyte hypertrophy) did not affect WARP expression in the cardiomyocytes (Fig 2E). WARP expression levels also significantly correlated with aSMA and COL1 expression levels in cardiac fibroblasts *in vitro*, however these correlations were less strong as compared to our *in vivo* results, as shown by the lower R^2^ values (Fig 2F and 2G). Since WARP and bFGF have been shown to interact with the same domain on perlecan [13], and since bFGF is a known inhibitor of fibroblast to myofibroblast conversion [20, 28], we investigated the effect of WARP on the extracellular and intracellular bFGF protein levels in cardiac fibroblasts. siRNA against WARP caused an 88% reduction of WARP gene expression in un-stimulated cells and a 90% reduction in stimulated cells (data not shown). TGFβ stimulation in scrambled control siRNA treated fibroblasts resulted in extracellular bFGF protein levels significantly decreasing (Fig 2H), whilst intracellular bFGF levels did not change (Fig 2I). WARP knockdown alone did not affect extra-or intra-cellular bFGF levels. However, when cells were stimulated with TGFβ (and WARP knocked down) there was no significant decrease in extracellular bFGF levels anymore (Fig 2H). Furthermore intracellular bFGF levels in the WARP-siRNA treated cells were increased as compared to the un-stimulated WARP siRNA treated cells and as compared to the stimulated scrambled siRNA treated cells (p = 0.08) (Fig 2I). Interestingly, WARP knockdown also resulted in a decrease in aSMA levels (1.05±0.25 in the control siRNA group versus 0.23±0.05 in the WARP siRNA group, p = 0.001), but not in COL1 levels (1.06±0.05 in the control siRNA group versus 1.13±0.10 in the WARP siRNA group). Overall, these data demonstrate that WARP expression correlates with markers of fibroblast to myofibroblast transition and indicate that WARP may influence the transition of fibroblasts into myofibroblasts by affecting bFGF levels.

### In-house bred Australian C57Bl/6-J WARP KO mice are protected from cardiac rupture and adverse infarct healing

**Fig 3:**
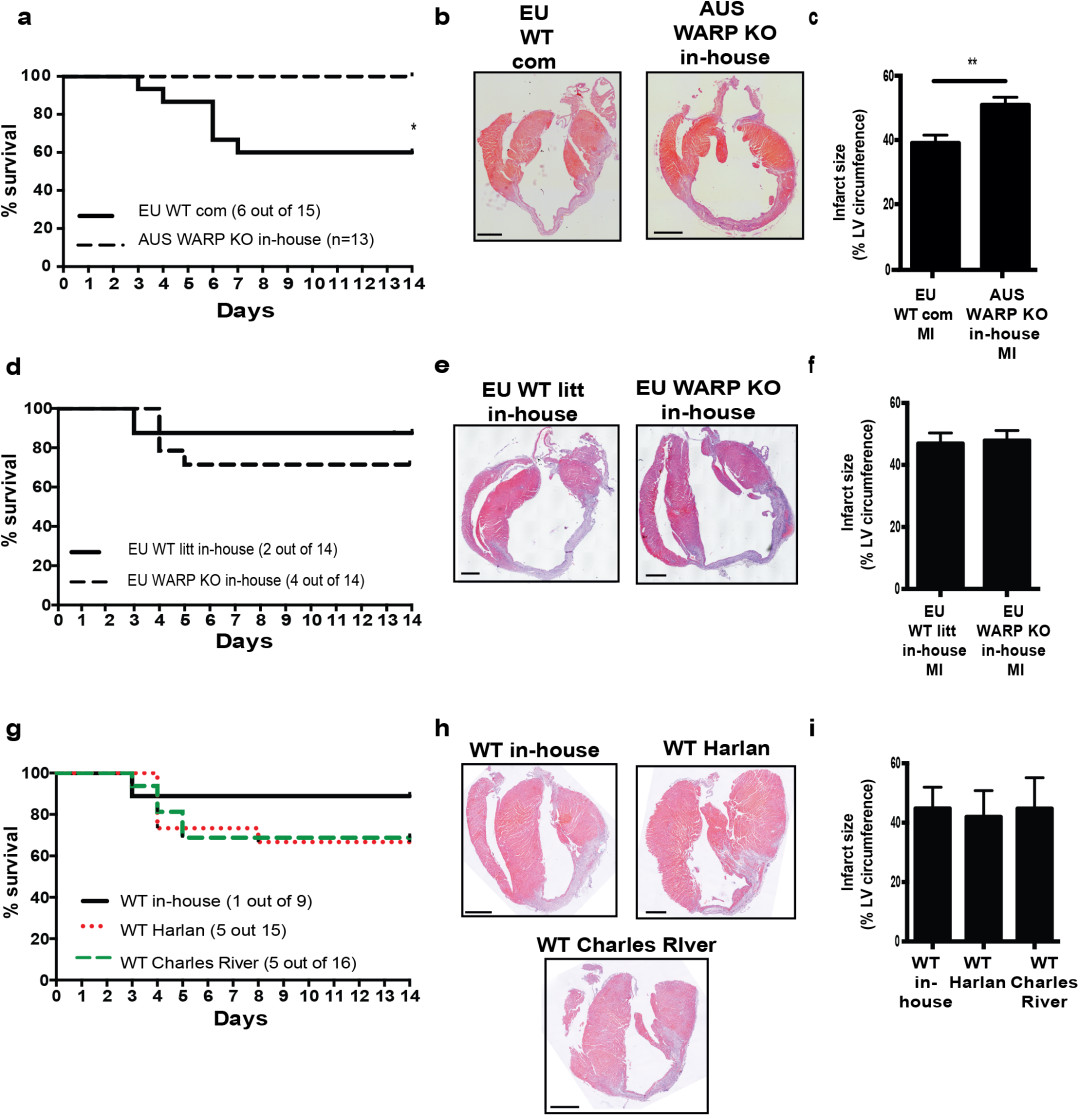
Genetic background, and not the absence of WARP, determines predisposition to cardiac rupture. (a) Improved survival in in-house bred Australian (AUS) WARP KO mice compared to commercial (com) European (EU) WT mice after an MI is shown by a Kaplan-Meier curve. (b & c) Histological analysis of LV sections of infarcted hearts (hematoxylin-eosin stained) revealed larger infarct sizes in in-house bred AUS WARP KO mice compared to EU WT com mice. (d) There was no difference in survival anymore when AUS WARP KO mice were backcrossed to the European C57Bl/6-J background and all mice were bred in-house. (e & f) Infarct sizes are the same after mice were backcrossed to the European C57Bl/6-J background. (g) Breeding/housing influences predisposition to cardiac rupture post-MI. WT mice bred in-house show improved survival after infarction when compared to commercial WT mice purchased from 2 different providers: Harlan and Charles River. (h&i) Despite the difference in survival, no difference in infarct size was seen between in-house bred and commercially purchased WT mice. n≥3; *p<0.05; **p<0.01; bars 1000 μm.

To further elucidate the role of WARP in cardiac remodeling after MI, we used the mouse model of permanent coronary occlusion in mice lacking WARP. WARP KO mice were backcrossed more than 10 times on a C57Bl/6-J background in Australia, imported to Europe, bred in-house, and subjected to the ligation of the left descending coronary artery in parallel with commercial WT mice of the same C57Bl/6-J background, but of European source. In-house bred Australian WARP KO mice showed significant improved survival as compared to commercial European WT mice 14 days after MI: 100% of the in-house bred Australian WARP KO mice (n = 13) survived while 40% of the commercial European WT mice (6 out of 15) died as a result of cardiac rupture (Fig 3A). There were no differences in heart weight to body weight or lung weight to body weight ratios (Table 1). This improved survival was not caused by smaller or thinner infarcts in the in-house bred Australian WARP KO mice as histological analysis of the infarcted areas revealed slightly larger infarct sizes in the WARP KO mice (Table 1 and Fig 3B and 3C), which were equal in thickness (Table 1 and Fig 3B). In the in-house bred Australian WARP KO group, the infarcts contained more viable cells compared to the commercial European WT mice (Table 1 and Fig 3B). Leukocyte infiltration or capillary density, as measured by the amount of CD45 positive leukocytes and CD31 positive capillaries in the remote and infarcted areas (Table 1), did not differ and could therefore not explain the improved cell viability in the infarcts of in-house bred Australian WARP KO mice. During proper infarct healing, collagen matures, and the fraction of thick, tightly cross-linked orange-red collagen fibers gradually replace the loosely assembled thin yellow-green fraction of collagen fibers [5, 29]. Despite a significant difference in mortality due to cardiac rupture, analysis of Picro Sirius Red stained sections showed no difference in total collagen content in the infarcted areas between the two groups (Table 1). Furthermore, there was no difference in collagen maturation, as shown by the equal fractions of orange-red and yellow-green collagen fibers in the infarcts of in-house bred Australian WARP KO and the commercial European WT mice (Table 1).

**Table 1:** Morphological and histological analysis in surviving commercial EU WT and in-house bred AUS WARP KO mice.

	**Sham**		**MI**	
	**EU WT com (n = 4)**	**AUS WARP KO in-house (n = 4)**	**EU WT com (n = 9)**	**AUS WARP KO in-house (n = 13)**
**HW / BW (mg/g)**	4.27 ±0.12	3.94 ± 0.18	4.75 ± 0.14	4.80 ± 0.26
**LW / BW (mg/g)**	6.56 ± 0.58	6.41 ± 0.76	6.64 ± 0.44	6.26 ± 0.34
	**Remote**		**Infarct**	
	**EU WT com (n = 5)**	**AUS WARP KO in-house (n = 7)**	**EU WT com (n = 5)**	**AUS WARP KO in-house (n = 7)**
**Infarct size (%)**	N.A.	N.A.	39.10 ± 2.433	50.93 ± 2.39^##^
**Infarct thickness (A.U.)**	N.A.	N.A.	100.00 ± 19.50	79.70 ± 4.44
**Viable area (%)**	N.A.	N.A.	15.45 ± 0.60	29.29 ± 4.06^##^
**CD45 count (cells/mm** ^**2**^ **)**	26 ± 5	60 ± 20	274 ± 36**	200 ± 55
**CD31 count (vessels/mm** ^**2**^ **)**	1707 ± 271	2389 ± 456	118 ± 27**	169 ± 58***
**Collagen deposition (%)**	N.A.	N.A.	61.17 ± 4.12	52.75 ± 3.24
**Fraction O-R thick/Y-G thin collagen fibers**	N.A.	N.A.	0.5 ± 0.05 / 0.5 ± 0.05	0.4 ± 0.05 / 0.6 ± 0.05

MI indicates myocardial infarction; EU European; AUS Australian; WT wild type; KO knock out; WARP Von Willebrand A domain related protein; com commercial; HW heart weight; BW body weight; LW lung weight; N.A. not applicable; A.U. arbitrary units; mm millimeter.

^##^p≤0.01 WT vs KO Infarct

**p≤0.01

***p≤0.001 Remote vs Infarct.

### Genetic background, and not the absence of WARP, determines predisposition to cardiac rupture

The Australian WARP KO mice were backcrossed on a European C57Bl/6-J background (three times) and a myocardial infarction study was performed with these “Europeanized” WARP KO mice and their WT littermates, with all of them bred in-house. There were no significant differences in mortality between KO and WT mice anymore: 29% of the “Europeanized” WARP KO (4 out of 14) and 13% of the WT littermates (2 out of 14) died due to cardiac rupture (Fig 3D). Moreover, while the “Europeanized” WARP KO mice did worse after MI, the WT mice had improved survival due to the crossbreeding. In the mice surviving MI, no difference in heart weight to body weight or lung weight to body weight ratios was seen (Table 2), and infarct size (Table 2 and Fig 3E and 3F) and thickness (Table 2 and Fig 3E) were equal in both groups. Furthermore, the amount of viable cells in the infarcted area and the amount of leukocytes, and capillary density in remote and infarcted tissue did not differ between both genotypes (Table 2 and Fig 3E). There was no difference in total collagen content and collagen maturation in the infarcts of the “Europeanized” WARP KO mice and WT littermates (Table 2). Finally, systolic function, as measured by echocardiography, was not different between the two groups (Table 3).

**Table 2:** Morphological and histological analysis in in-house bred EU WT litt and EU WARP KO mice.

	**Sham**		**MI**	
	**EU WT litt in-house (n = 5)**	**EU WARP KO in-house (n = 7)**	**EU WT litt in-house (n = 12)**	**EU WARP KO in-house (n = 10)**
**HW / BW (mg/g)**	3.79 ± 0.22	4.10 ± 0.08	5.29 ± 0.26^##^	5.71 ± 0.37^##^
**LW / BW (mg/g)**	6.22 ± 0.32	6.69 ± 0.25	8.20 ± 1.12	7.66 ± 0.79
	**Remote**		**Infarct**	
	**EU WT litt in-house (n≥4)**	**EU WARP KO in-house (n≥6)**	**EU WT litt in-house (n≥4)**	**EU WARP KO in-house (n≥6)**
**Infarct size (%)**	N.A.	N.A.	47.96 ± 4.91	47.88 ± 3.22
**Infarct thickness (A.U.)**	N.A.	N.A.	100.00 ± 10.73	93.56 ± 10.85
**Viable area (%)**	N.A.	N.A.	18.03 ± 1.91	11.36 ± 3.30
**CD45 count (cells/mm** ^**2**^ **)**	58 ± 15	90 ± 16	147 ± 8*	155 ± 69
**CD31 count (vessels/mm** ^**2**^ **)**	1769 ± 73	1899 ± 141	86 ± 21***	81 ± 22***
**Collagen deposition (%)**	N.A.	N.A.	50.8 ± 3.4	51.8 ± 3.3
**Fraction O-R thick/Y-G thin collagen fibers**	N.A.	N.A.	0.4 ± 0.04 / 0.6 ± 0.04	0.4 ± 0.07 / 0.6 ± 0.07

MI indicates myocardial infarction; EU European; WT wild type; KO knock out; WARP Von Willebrand A domain related protein; com commercial; litt littermate; HW heart weight; BW body weight; LW lung weight; N.A. not applicable; A.U. arbitrary units; mm millimeter.

^##^p≤0.01sham vs MI

*p ≤ 0.05

***p≤0.001 Remote vs Infarct.

**Table 3:** Functional analysis in in-house bred EU WT litt and EU WARP KO mice.

	Baseline		14 days post-MI	
	EU WT litt in-house (n = 8)	EU WARP KO in-house (n = 16)	EU WT litt in-house (n = 5)	EU WARP KO in-house (n = 7)
**EF (%)**	40.32 ±1.87	41.02 ± 3.14	27.01 ± 3.55*	24.37 ± 1.83***
**LVIDd (mm)**	3.63 ± 0.12	3.71 ± 0.09	5.06 ± 0.38***	5.41 ± 0.25***
**LVIDs (mm)**	2.76 ± 0.13	2.83 ± 0.11	4.45 ± 0.44***	4.68 ± 0.31***
**PWd (mm)**	0.65 ± 0.03	0.67 ± 0.02	0.60 ± 0.05	0.68 ± 0.06
**IVSd (mm)**	0.76 ± 0.05	0.83 ± 0.08	0.72 ± 0.05	0.81 ± 0.05
**HR (bpm)**	554 ± 8	563 ± 13	517 ± 22	600 ± 52

MI indicates myocardial infarction; EU European; WT wild type; KO knock out; WARP Von Willebrand A domain related protein; com commercial; litt littermate; EF ejection fraction; LVIDd left ventricular internal diameter diastolic; LVIDs left ventricular internal diameter systolic; PWd posterior wall diastolic; IVSd interventricular septum diastolic; HR heart rate; mm millimeter; bpm beats per minute.

*p≤0.05

***p≤0.001 in baseline vs MI.

Interestingly, in the in-house bred WT colony littermates a different response post-MI was seen when compared with the commercially sourced WT mice, with less cardiac rupture (29% vs 40% respectively) and slightly bigger infarct sizes (48% vs 39% respectively). This might not be solely related to the difference in continental origin and consecutive crossbreeding, but also to housing conditions, experimental variation or single nucleotide polymorphisms (SNPs) [30]. Therefore we compared the cardiac response following MI in our in-house colony WT mice with C57BL/6-J WT mice purchased from Harlan and also with C57BL6J WT mice purchased from Charles River, which have the same SNPs as Australian C57BLJ mice.

Commercial WT mice (from both Harlan and Charles River) continued to show higher mortality 14 days after MI: 33% of the commercial WT mice from Harlan (5 out of 15) and 31% of the commercial WT mice from Charles River (5 out of 16) died while only 11% of the in-house bred WT mice (1 out of 9) died as a result of cardiac rupture (Fig 3G). There were no differences in heart weight to body weight or lung weight to body weight ratios (Table 4). The improved survival was not related to smaller or thinner infarcts as histological analysis of the infarcted areas revealed similar infarct sizes in all the WT mice (Table 4 and Fig 3H and 3I), which were equal in thickness (Table 4 and Fig 3H).

**Table 4:** Morphological and histological analysis of surviving infarcted WT mice bred in-house and of commercial source.

	WT in-house (n = 8)	WT Harlan (n = 10)	WT Charles River (n = 11)
**HW / BW (mg/g)**	5.08 ± 0.30	4.88 ± 0.18	4.63 ± 0.15
**LW / BW (mg/g)**	8.92 ± 1.51	7.59 ± 0.31	8.69 ± 1.08
**Infarct size (%)**	44.79 ± 4.11	41.93 ± 3.13	44.72 ± 4.26
**Infarct thickness (A.U.)**	100 ± 5.18	102.39 ± 12.74	91.32 ± 7.41

HW heart weight; BW body weight; LW lung weight; A.U. arbitrary units.

## Discussion

We hypothesized that WARP plays a role in the wound healing response following MI as WARP expression was induced at 3 and 7 days after MI, in association with TGFβ-induced myofibroblast transformation [20, 28]. WARP expression correlated with aSMA and COL1 expression, 2 markers of fibroblast to myofibroblast transition, *in vivo and in vitro*, and WARP reduced extra- and intracellular bFGF, a growth factor shown to be protective in cardiac remodeling after MI [19]. We therefore assessed the role for WARP during cardiac remodeling in an MI model using in-house bred Australian C57Bl/6-J WARP KO mice and commercial European WT C57Bl/6-J mice. The results indicated a crucial role for WARP in cardiac remodeling as a consequence of ischemic heart disease yet subsequent experiments using “Europeanized” WARP KO and their WT littermates, all of them bred in-house, revealed that in fact the continental/breeding/housing origin was responsible for the previously seen protective effect in the WARP KO mice. In line, differences were observed in the commercial sourced WT mice and the in-house colony bred WT mice. Thus the breeding strategy of the mice, the continental and breeding origin in combination with the housing conditions, determined the rupture incidence and not the absence of WARP.

Earlier research already addressed the importance of the genetic background in susceptibility to CVD and exercise induced cardiac function and remodeling [24, 25, 31–33]. Van den Borne and colleagues studied the MI model in 5 different mouse strains (BalbC, C57Bl/6, FVB, 129S6, and Swiss) in order to determine which mouse strain is the best choice to study different aspects of ischemic heart disease. They concluded that the 129S6 mouse is best to study infarct rupture, while BalbC and Swiss mice are better models to study infarct thinning after MI [34]. Research done by Gorog and colleagues compared commercial outbred mice, i.e. mice that are maintained as closed colonies of genetically-variable composition, with WT offspring of 2 in-house bred heterozygous colonies of the same C57Bl/6-J strain and showed a difference in susceptibility to global ischemia in Langendorff-perfused hearts [35]. Although now most researchers are aware of the importance of the genetic background of the inbred strain used in the generation of transgenic and knockout mice, there is insufficient recognition of the regional differences and substrain-variability that influences susceptibility to CVD in mice. Within the C57Bl/6-J strain, there are genetic differences, which could be responsible for such a phenotype like we see in this study. Several distributors of the C57Bl/6-J strain exist, where breeding stocks are kept separate, allowing the accumulation of genetic differences due to genetic drift and individual variability. Recently, Zurita and colleagues and Mekada and colleagues described genetic polymorphisms among C57Bl/6 strains [23, 26]. Among 1449 SNPs investigated, C57Bl/6-J mice purchased from Harlan differ at 3 SNPs when compared to C57Bl/6-J mice purchased from Australia or from Charles River [23]. Of these 3 SNPs, 1 is associated with the Naaladl2 (N-acetylated alpha-linked acidic dipeptidase-like 2) gene. This gene has not yet been annotated in mice and hence has no known molecular function. The 2 other SNPs are not associated with genes (information found at the SNPs collection of the Mouse Genome Database, phenome.jax.org/SNP/, at the Mouse Genome Informatics, www.informatics.jax.org/marker, and at ensembl.org). It is however unlikely that SNPs are responsible for the differences in rupture rates post MI seeing as mortality rates were similar in WT mice purchased from Harlan and from Charles River.

The use of genetically manipulated mice is a widespread tool to study the effects of a specific gene in models of cardiovascular disease (CVD). Thanks to intercontinental collaborations, air travel is commonplace these days for genetically manipulated mice, with numerous labs breeding their KO mice in-house and purchasing the WT mice from a commercial source. Unfortunately, the literature poorly describes breeding strategies, genetic backgrounds or the continental source of mice studied, thereby making it difficult to estimate how many studies have mistakenly attributed a phenotype. This study highlights the importance of implementing a breeding strategy that takes such factors into consideration, stresses the importance of using the correct littermate controls and conveying this information in publications.

In summary, this study indicates a putative function for WARP in the cardiac fibroblast to myofibroblast transition, yet a redundant role in the acute healing process following MI. Finally, more importance should be paid to the continental and especially housing/breeding origin of mice used in research and may be another argument to reduce the use of animals in cardiovascular research.

## Materials and Methods

### *In vitro* experiments in neonatal rat cardiac fibroblasts and myocytes

Cardiac fibroblasts and myocytes were isolated by enzymatic disassociation from 2-day-old neonatal Lewis rats as previously described [36]. The Animal Care and Use Committee of the University of Leuven approved the described study protocols. Experiments were performed according to the official rules formulated in the Belgian law on the care and use of experimental animals and all efforts were made to minimize suffering (License number 161/2011). For experiments, second-passage cardiac fibroblasts (70–90% confluent) and freshly isolated cardiomyocytes in 6-well plates were used. The cardiac fibroblasts were maintained in medium (DMEM 22320, Invitrogen) supplemented with 10% fetal bovine serum (FBS) and 1% penicillin-streptomycin and the cardiomyocytes were maintained in medium (80% DMEM 11966, 20% M199, Invitrogen) supplemented with 0.2% glucose, 0.125% gentamycin, 1.25% penicillin-streptomycin and 10% bovine serum albumin. Both cell types were incubated at 37°C in a humidified chamber. The day before an experiment, the cardiac cells were starved overnight in DMEM without antibiotics or serum. After starvation, cardiomyocytes were stimulated with ET-1 (10nM), for 24 hours and thereafter supernatants were collected and cells were lysed and collected in radioimmunoprecipitation assay (RIPA) buffer containing 2% phosphatase-inhibitors and 4% protease-inhibitors for protein isolation or in RLT buffer (QIAGEN) containing 1% β-mercapto-ethanol for RNA isolation. After starvation of the cardiac fibroblasts, WARP mRNA expression was reduced, by treatment of the cells with siRNA against WARP (Invitrogen) for 4 hours, or with negative control siRNA (Invitrogen) for control samples. Equal volumes of siRNA and lipofectamine (Invitrogen) were mixed 1/50 in Optimem (Invitrogen) and 500 μl of this mixture was added to the 1.5 ml of medium on the cells. After 4 hours, the medium of the cardiac fibroblasts was diluted 1/1 with DMEM containing 0.5% FBS and 1% penicillin-streptomycin. The next day, the medium was refreshed and 4 hours later the fibroblasts were treated with 1 ng/ml transforming growth factor β (TGFβ) (PeproTech) for 24 hours. Supernatants and cells were collected as described for cardiomyocytes.

### Mouse models

The Animal Care and Use Committee of the University of Leuven approved all described study protocols. Experiments were performed according to the official rules formulated in the Belgian law on the care and use of experimental animals (License number 121/2008). All surgery was performed under ketamine and xylazine anesthesia at a dose of 100 mg/kg and 10 mg/kg respectively, and all efforts were made to minimize suffering. Experimental MI and sham operation were performed as previously described [37]. Briefly, animals were anesthetized, fixed in the supine position and after tracheal intubation, positive pressure respiration was initiated (MiniVent Ventilator; Harvard Apparatus). Pectoral muscles were dissected and retracted. Left thoracotomy was performed in the fourth intercostal space and after opening of the pericardium, the left anterior descending artery was ligated. Infarction was evident from discoloration of the distal myocardial tissue. After layered closure of intercostal muscles and skin, the animals recovered at 37°C. Sham-operated animals were subjected to similar surgery but no ligature was placed. Death within 24h was considered a complication of surgery or due to cardiogenic shock (fatal MI); these animals (< 5%) were excluded from further analysis. Only animals with infarct sizes > 25% were included in the final analysis. WT C57Bl/6-J mice were purchased from Harlan (Europe), Charles River (Europe), and WARP KO C57Bl/6-J mice were kindly provided by professor JF Bateman. These WARP KO C57Bl/6-J mice resulted from heterozygous breeding of WARP +/- mice, termed Vwa1+/-, which were produced under contract by Ozgene Pty Ltd., Western Australia and backcrossed on a C57Bl/6-J background more than 10 times. For an MI time series 18 WT mice purchased from Harlan on the European C57Bl/6-J background of 7 to 13 weeks old were used and mice were sacrificed after 3, 7 or 14 days. For the first MI study, 20 WT mice of commercial European C57Bl6/J origin (Harlan) and 20 WARP KO mice of Australian origin, bred in-house, (7 to 13 weeks) were used; all experiments were performed using gender and age-matched mice and mice were sacrificed after 14 days. Next, WARP KO mice were backcrossed 3 x on the European C57Bl/6-J background and bred heterozygous in-house. A new MI study using 21 WARP KO mice and 21 WT littermates (9 to 15 weeks) was performed. MI and sham operation were performed as in the first experiments and mice were sacrificed after 14 days. Finally, an MI study using 9 in-house bred WT mice, 15 WT mice purchased from Harlan and 16 WT mice purchased from Charles River (6 to 12 weeks) was performed as in the first experiments and mice were sacrificed after 14 days. In all experiments, hearts and lungs were removed, and hearts were prepared for molecular and histological analysis. Humane endpoints were used during all experiments: animals were monitored daily for grade of activity, healing of the surgical wound, weight loss, normal breathing and absence of ruffled fur, but no mice needed to be sacrificed prior to experimental endpoints. Mice that died during the experiment, died suddenly from cardiac rupture.

### Histology and microscopy

Cardiac tissue was processed and histochemical and immunohistochemical analyses were performed as previously described [37–39], and all morphometric analyses were done on midsagittal sections. Hematoxylin and eosin–stained sections (4 μm) were used to assess the infarct size, infarct thickness and residual viable area. Percentage of infarct size was expressed as the fractional circumference of the infarcted versus infarcted plus non-infarcted left ventricle (LV) wall and septum, which was assessed by measuring the midline circumference of the LV. Infarct thickness was measured as the mean of 12 measurements across the infarcted LV wall. Residual viable area was determined as the percentage of the total infarcted area. The number of CD-45 –staining cells (monoclonal rat antibody, BD, 553076, clone 30-F11, 5μg/ml) and CD-31 –staining capillaries (monoclonal rat antibody, BD, 557355, clone MEC13.3, 1μg/ml) in the infarct zone was measured per mm². For colocalization studies, sections were stained for WARP (polyclonal goat antibody, R & D Systems, AF4927, antigen NSO-derived rmWARP isoform1, 2μg/ml), perlecan (monoclonal rat antibody, Millipore, MAB19480, clone A7L6, 1μg/ml), CD-45 (monoclonal rat antibody, BD, 553076, clone 30-F11, 5μg/ml) and alpha smooth muscle cells (monoclonal mouse antibody, Sigma, C6198, clone 1A4, 1μg/ml) and subsequently incubated with Biotin-labeled secondary antibodies followed by amplification with the signal amplification system (streptavidin-HRP-C-fluorescein/Cy3; PerkinElmer). Nuclei were stained with DAPI (Invitrogen). To assess the amount of the newly formed collagen matrix, Picro Sirius Red staining was performed as previously described [39, 40]. Microscopic analyses were performed using a microscope (Leitz DMRXE; Leica), and QWin morphometry software (Leica). Confocal microscopy was performed on a Zeiss CLSM 510 Meta NLO microscope (Leica) using the Zen software (Leica). All analyses were performed according to standard operating procedures.

### RNA isolation and expression

RNA was isolated from sham and infarcted tissue or cell lysates with the RNeasy Mini kit (QIAGEN) according to the manufacturer’s guidelines and was stored at -80°C. RNA was reverse transcribed into complementary DNA with the iScript cDNA synthesis kit (BioRad) according to the manufacturer’s instructions. Real-time quantitative PCR was performed with SYBR green PCR Master mix (Applied Biosystems). Primers were designed with primer-BLAST (NCBI) and built to contain an intron- exon boundary. Primers were designed for mouse WARP (5’GATCTTCCTATCATTGCCCG3’; 5’AAGCCACTGGACAGAACCTC3’), rat WARP (5’AGCTCCGGCTGAGAAGCACCT3’; 5’GGCTGCATCGCATCAATAATGGCAC3’), mouse aSMA (5’GTCCCAGACATCAGGGAGTAA3’; 5’TCGGATACTTCAGCGTCAGGA3’), rat aSMA (5’GTCCCAGACACCAGGGAGTGA3’; 5’TCGGATACTTCAGGGTCAGGA3’), mouse COL1 (5’CTTCACCTACAGCACCCTTGTG3’; 5’ CTTGGTGGTTTTGTATTCGATGAC3’), rat COL1 (5’ CCGCCCGCACATGC3’; 5’ CTCCATGTTGCAGTAGACCTTGAT3’), and mouse GAPDH (5’GGTGGACCTCATGGCCTACA3’; 5’TCGTTCCTGTGACTCGTTCTCTC3’) or rat GAPDH (5’GGTGGACCTCATGGCCTACA3’; 5’TCGTTCCTATGACTCTCGTTCTCTC3’) were used as housekeeping gene.

### Western Blotting

Proteins in sham, remote, and infarcted tissue were separated by SDS-PAGE and were subsequently immunoblotted for the detection of WARP (polyclonal goat antibody, R&D systems, AF4927, antigen NSO-derived rmWARP isoform1, 0.2μg/ml) and GAPDH (monoclonal mouse antibody, Fitzgerald, 10R-G109a, clone 6C5, 0.1μg/ml) overnight at 4°C. Signals were visualized using Hyperfilm ECL (Amersham Biosciences) and quantified using Image J software. Protein levels were expressed relative to protein levels of GAPDH.

### bFGF- ELISA

To release and quantify extracellular bFGF in fibroblast cultures, the supernatants were removed and fibroblasts were incubated with 0.25 ml of 20 mM Tris-HCl (pH 7.2) and 2 M NaCl for 2 min as previously described [41]. The bFGF in this medium was quantified using a bFGF detection ELISA kit (R&D Systems, DFB50) according to the manufacturer’s instructions. The fibroblasts remaining were lysed and collected in RIPA buffer as described above and used to quantify intracellular bFGF in the same ELISA as described above.

### Echo analysis

Mice were anesthetized (2% isoflurane, ecuphar) and echocardiograpy was performed at day 0 and day 14 by transthoracic echocardiography with a 13-MHz transducer (i13L, GE ultrasound; Horton Norway) on a Vingmed Vivid 7 scanner (GE ultrasound, Horton, Norway). Heart rate (HR) and LV diameters at end-diastole (LVIDd), end-systole (LVIDs), septal wall thickness (ISd), LV posterior wall thickness in end diastole (PWd), were measured, and ejection fraction (EF) was calculated.

### Statistical analysis

Data were expressed as the mean ± SEM. For echocardiographic measurements repeated measures were performed. Histological and molecular analyses in sham-operated and infarcted groups were performed in independent groups. Normal distribution of all continuous variables was tested according to the method of Kolmogorov and Smirnov. An unpaired Student’s t test for 2 groups or ANOVA, followed by a Bonferroni post hoc test for more groups was used in most of the comparisons when groups passed the normality test. When the standard deviation of two groups significantly differed, a Mann-Whitney test for 2 groups or a Kruskal-Wallis test, followed by a Dunn’s post hoc test for more groups, was used. For correlations, the Pearson correlation coefficient was calculated when variables passed normality test, or a Spearman correlation coefficient was calculated when the standard deviation of the groups significantly differed. A paired Student’s t test was used to analyze baseline and follow-up echocardiographic measurements. The survival curve after MI was obtained by the Kaplan-Meier method and compared by the log-rank test. A two-sided p-value of ≤ 0.05 was considered statistically significant.

## Supporting Information

S1 Fig Original unadjusted blots for WARP expression pattern in the heart. (TIF)
