# Supplementary figures and images for: Breeding Strategy Determines Rupture Incidence in Post-Infarct Healing WARPing Cardiovascular Research
Source: PLoS One. 2015 Sep 25;10(9):e0139199. doi: 10.1371/journal.pone.0139199 (PMC4583407; doi:10.1371/journal.pone.0139199)

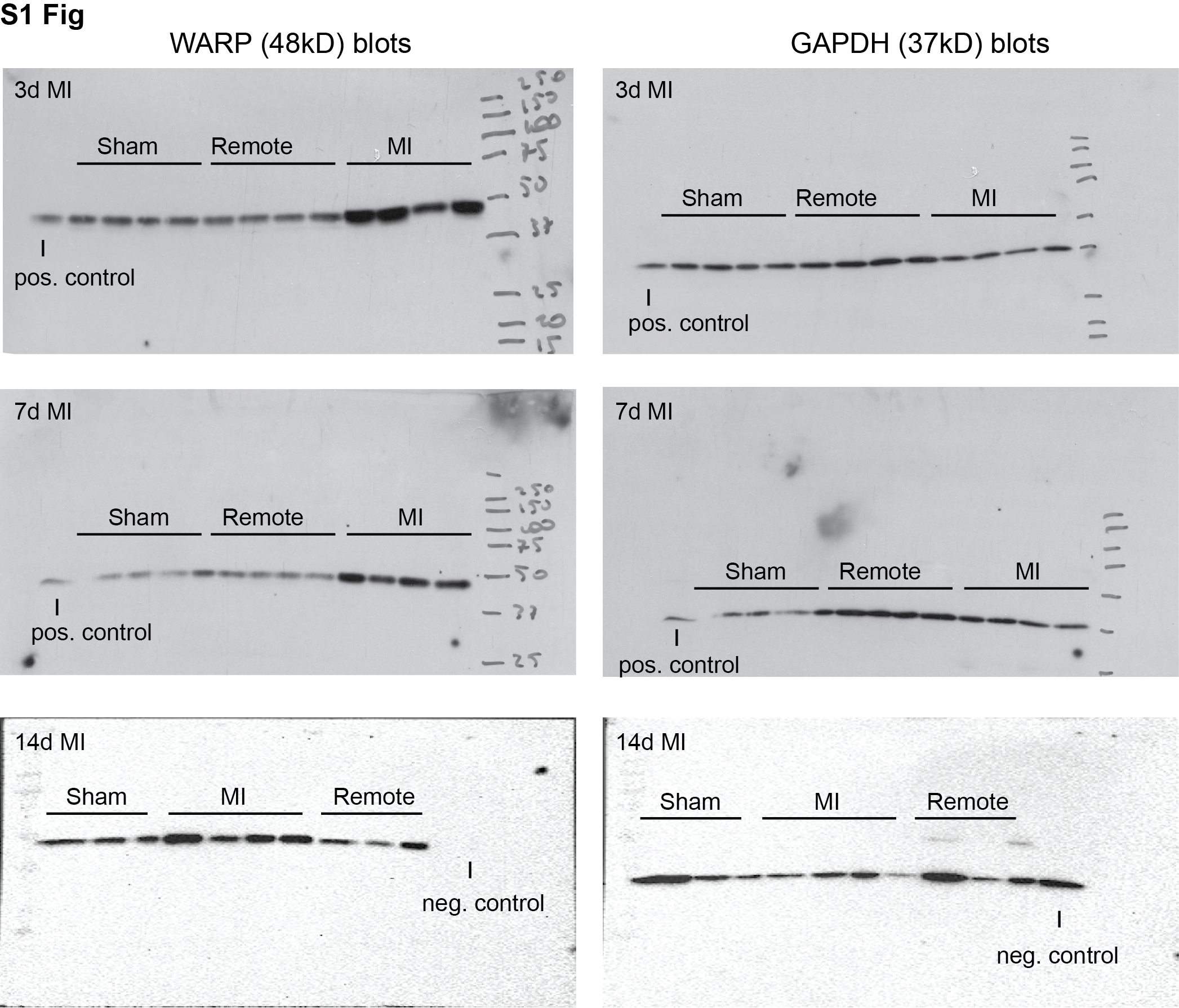

Supplement: S1 Fig — (TIF) [file pone.0139199.s001.tif]
